# Relationship between mitochondrial haplogroup and seasonal changes of physiological responses to cold

**DOI:** 10.1186/1880-6805-33-27

**Published:** 2014-09-03

**Authors:** Takayuki Nishimura, Shigeki Watanuki

**Affiliations:** 1 Department of Public Health, Nagasaki University Graduate School of Biomedical Sciences, Nagasaki, Japan; 2 Department of Human Science, Faculty of Design, Kyushu University, Fukuoka, Japan

**Keywords:** mtDNA haplogroup, NST, VO_2_, Cold exposure, Seasonal acclimatization

## Abstract

**Background:**

Physiological responses to cold exhibit individual variation that can be affected by various factors, such as morphological characteristics, seasonal changes, and lifestyle; however, the genetic factors associated with this variation remain unclear. Recent studies have identified mtDNA as a potential genetic factor affecting cold adaptation. In addition, non-shivering thermogenesis (NST), a process closely related to mitochondrial dynamics, has also been suggested as an important factor affecting human response to cold. The present study aimed to clarify the relationship between mitochondrial haplogroup and NST during periods of mild cold exposure.

**Methods:**

Seventeen healthy university students (D: n = 8, non-D: n = 9) participated in the present study during summer and winter. A climate chamber was programmed so that ambient temperature inside dropped from 28°C to 16°C over the course of an 80-minute period. Physiological parameters were recorded throughout the course of the experiments.

**Results:**

Increases in VO_2_ were significantly greater during periods of cold exposure in winter than they were during periods of cold exposure in summer, and individuals from the D group exhibited greater winter values of ΔVO_2_ than individuals from the non-D group.

T_re_ was significantly lower during periods of rest and cold exposure in winter; however, no significant difference was observed between T_re_ values of individuals in the D and non-D groups. In addition, although ${\bar{T}}_{\mathrm{dist}}$ was significantly lower during periods of rest in winter than it was during those same periods in summer, no significant seasonal differences in values of ${\bar{T}}_{\mathrm{dist}}$ were observed during periods of cold exposure.

**Conclusions:**

Results of the present study indicated that NST was greater in winter, and that the D group exhibited greater NST than the non-D group during winter. Despite the differences between groups in NST, no significant differences in rectal and skin temperatures were found between groups in either season. Therefore, it was supposed that mitochondrial DNA haplogroups had a greater effect on variation in energy expenditure involving NST than they had on insulative responses. Future studies are necessary in order to investigate more multiple candidate genes related to human cold adaptation and to elucidate the relationship between gene polymorphism and physiological polytypism.

## Background

*Homo sapiens* originated in Africa approximately 160,000 year ago, after which time the species rapidly spread around the world during the last glacial period (110,000 to 12,000 years ago) [1]. Our ancestors adapted to various environments during this migration, with both cultural and physiological adaptations proving necessary in order to survive in cold environments [2]. Physiological adaptations to cold, such as the metabolic adaptation of the Inuit [3] and the insulative adaptation of Australian aborigines [4], are well known. Adaptations such as these might have involved genetic adaptations, since these groups were settled in their respective environments for long periods of time. On the other hand, individual variations in both metabolic and insulative type with respect to cold stimuli have also been reported as physiological polytypism within populations [5].

In urban residents, physiological responses to cold environments include vasoconstriction, which occurs rapidly in response to cold exposure in order to decrease heat loss; however, the range to which the thermal environment can be adjusted by vasoconstriction alone is narrow, and thermogenesis is typically required to maintain optimal body temperature. Thermogenesis can be divided into shivering thermogenesis (ST) and non-shivering thermogenesis (NST). These physiological responses to cold are affected by various environmental or individual factors such as season [6-8], lifestyle [9], and physical characteristics [10]. Recently, NST via metabolism of free fatty acids (FFA) by brown adipose tissue (BAT) has been determined to be an important source of metabolic heat in cold environments [11]. Furthermore, NST activated by BAT was determined to be greater either in winter [12] or after cold acclimatization [13]. Although genetic factors must exist, few studies examining the effects of genetic factors on physiological responses to cold have been undertaken.

In order to examine genetic factors, the present study focused on mitochondria and the mitochondrial genome. Mitochondria are organelles in the cell that generate ATP and heat by OXFOS (oxidative phosphorylation); this heat is the main source of heat contributing to human body temperature [14]. Furthermore, mitochondria play an important role in energy metabolism involving NST [14,15]. Mitochondria also possess their own genome (mtDNA), with mtDNA polymorphism having been used to help understand the origins of humanity and our moving history [1]. In addition, previous studies have suggested that mtDNA polymorphism was shaped by natural selection, especially in regions of cold climate, and that some haplogroups determined by mtDNA polymorphism had been specifically adapted to cold environments [14,16,17]. Previous studies have also indicated that various mtDNA haplogroups are related to maximum oxygen intake (VO_2max_) [18], athletic performance [19], and metabolic disease [20]. Results of studies such as these indicated that mtDNA haplogroups were associated with human cold adaptation, and that they affect energy expenditure in particular.

Despite the multitude of studies addressing the role of mtDNA haplogroups in human cold adaptation, no previous studies have directly evaluated cold tolerance. Our previous study aimed to examine the relationship between mtDNA haplogroup and physiological response to serve cold exposure (10°C) [21]. Results indicated that individuals of haplogroup D maintained higher core body temperatures in summer than individuals of haplogroup non-D, although no difference in body temperature could be detected between the two groups in winter. Haplogroup D tended to metabolize greater amounts of fat in winter than did haplogroup non-D, which indicated a greater level of NST in haplogroup D [21]. On the other hand, our previous experiment employed significant levels of cold exposure, which made it difficult to separate ST from NST as all participants shivered in both winter and summer. The present study aimed to clarify the relationship between mtDNA haplogroup and NST by employing periods of mild cold exposure during both summer and winter.

## Method

### Participants

Seventeen healthy university students (Japanese, male, 20 to 24 years old) who exhibited no clinical problems participated in the present study. After having the experimental procedure fully described to them, they consented to their participation in writing. The haplogroups of non-D subjects were M7 (4 participants), F (1 participants), B4 (3 participants), and N9a (1 participants). Table 1 shows the morphological characteristics of the D and non-D groups in each season. Body mass index (BMI) was calculated as follows:

**Table 1:** Participants’ morphological characteristics

**Season**	**Haplogroup**	**Height (cm)**	**Body mass (kg)**	**BMI**	**BSA (cm**^**2**^**)**	**Body fat (%)**
Summer	D (n = 8)	173.7 ± 7.4	60.8 ± 7.8	19.8 ± 2.2	1.71 ± 0.12	12.3 ± 2.2
	non-D (n = 9)	171.4 ± 8.6	57.6 ± 7.4	19.9 ± 2.0	1.68 ± 0.12	13.1 ± 2.5
Winter	D (n = 8)	173.4 ± 7.2	61.2 ± 6.4	20.3 ± 1.6	1.72 ± 0.11	12.8 ± 2.4
	non-D (n = 9)	170.8 ± 8.9	57.8 ± 7.6	19.8 ± 1.1	1.67 ± 0.12	13.6 ± 2.4

No significant differences in morphological characteristics were found between groups during summer and winter.

$$\mathrm{BMI}=\mathrm{Weight}\left(\mathrm{kg}\right)/\mathrm{Height}\left(m\right)2$$

Body surface area (BSA) was calculated using Krazumi’s Formula [22]. Experiments were performed with approval from the Ethics Committee of the Graduate School of Design, Kyushu University.

### DNA analysis

Total DNA was extracted from hair shafts by digestion in extraction buffer using ISOHAIR (Code Number 319-03401; Nippon Gene, Tokyo, Japan). The mtDNA spacer D-loop was amplified by PCR using primers M13RV-L15996 and M13(-21)-H408. The analyzed sequences of the D-loop primers were as follows:

mtDNA L15996, 5′-CTCCACCATTAGCACCCAAAGC-3′; and

mtDNA H408, 5′-CTGTTAAAAGTGCATACCGCCA-3′.

The thermocycling profile consisted of an initial denaturation step at 94°C for 1 minute, followed by 32 cycles of 30 seconds at 94°C, 30 seconds at 56°C, and 75 seconds at 72°C. Purified DNA was sequenced in both directions using an ABI PRISM 310 Genetic Analyzer (Applied Biosystems, Foster City, CA, USA) with a BigDye Terminator v3.1 Cycle Sequencing Kit (Applied Biosystems, Foster City, CA, USA).

### Study procedure

Experiments were conducted in summer (August to September) and winter (February to March) in Fukuoka, Japan. Average temperature in Fukuoka was 28.3°C in summer and 8.5°C in winter. Participants abstained from food and drink for a period of greater than two hours prior to entering the climate chamber. Various measurement sensors (temperatures sensors, gas analyzer) were attached to participants in an environment with a temperature of 28°C prior to experimentation for a period of 30 minutes. After participants had rested quietly for a period of 20 minutes after entering the climate chamber, ambient temperature within the climate chamber dropped from 28°C to 16°C over the course of an approximately 80-minute period. Parameters recorded were rectal temperature, skin temperature (at 7 locations), and oxygen intake during ‘rest time’ (0 to 20 minutes) and ‘cold exposure’ (20 to 100 minutes). An electromyogram and a subjective evaluation were also carried out.

Rectal temperature (T_re_) probes were inserted at a depth of 13 cm beyond the anal sphincter. Skin temperature sensors were attached with surgical tape to measurement sites on the forehead, abdomen, forearm, hand, thigh, leg, and foot. Measurements were taken at intervals of 10 seconds using a data logger (LT-8A, Gram Corporation, Saitama, Japan).

Skin temperature was calculated using the seven-point method of Hardy-DuBois [23]. Distal skin temperature ( ${\bar{T}}_{\mathrm{dist}}$) was derived using the following equation:

$$\begin{array}{l} {\bar{T}}_{\mathrm{dist}}=(0.14\;\times \;T_{\mathrm{forearm}}+\;0.05\;\times \;T_{\mathrm{hand}}+\;0.07\\ \;\times \;T_{\mathrm{foot}}+\;0.13\;\times \;T_{\mathrm{leg}})/0.39 \end{array}$$

Oxygen intake (VO_2_) and carbon dioxide output (VCO_2_) were measured using a respiratory gas analyzer (AE-300S, Minato Medical Science, Osaka, Japan) through a breathing tube with a Rudolph mask used to measure expired gas (Rudolph mask, Nihon Kohden, Tokyo, Japan). Respiratory exchange ratio (RER) was calculated as VCO_2_/VO_2_. Higher RER values indicated metabolism of glucose and lower RER values indicated metabolism of fat. Changes in the pectoralis major muscle were recorded using an electromyograph (PolyTele, Nihon Santeku, Kyoto, Japan). Data were recorded at a sampling frequency of 1000 Hz and a bandpass filter (20 to 500 Hz) was used in analysis.Electromyographic data obtained during cold exposure was based on muscular changes during the first ten minutes of rest time.

### Statistical analysis

Morphological data were compared by paired *t*-test. Physiological data were compared using three-way (haplogroup, season, and time) analysis of variance (ANOVA). All data were expressed as means ± standard error, and statistical significance was determined at *P* < 0.05.

## Results

### Changes in oxygen intake (ΔVO_2_)

**Figure 1:**
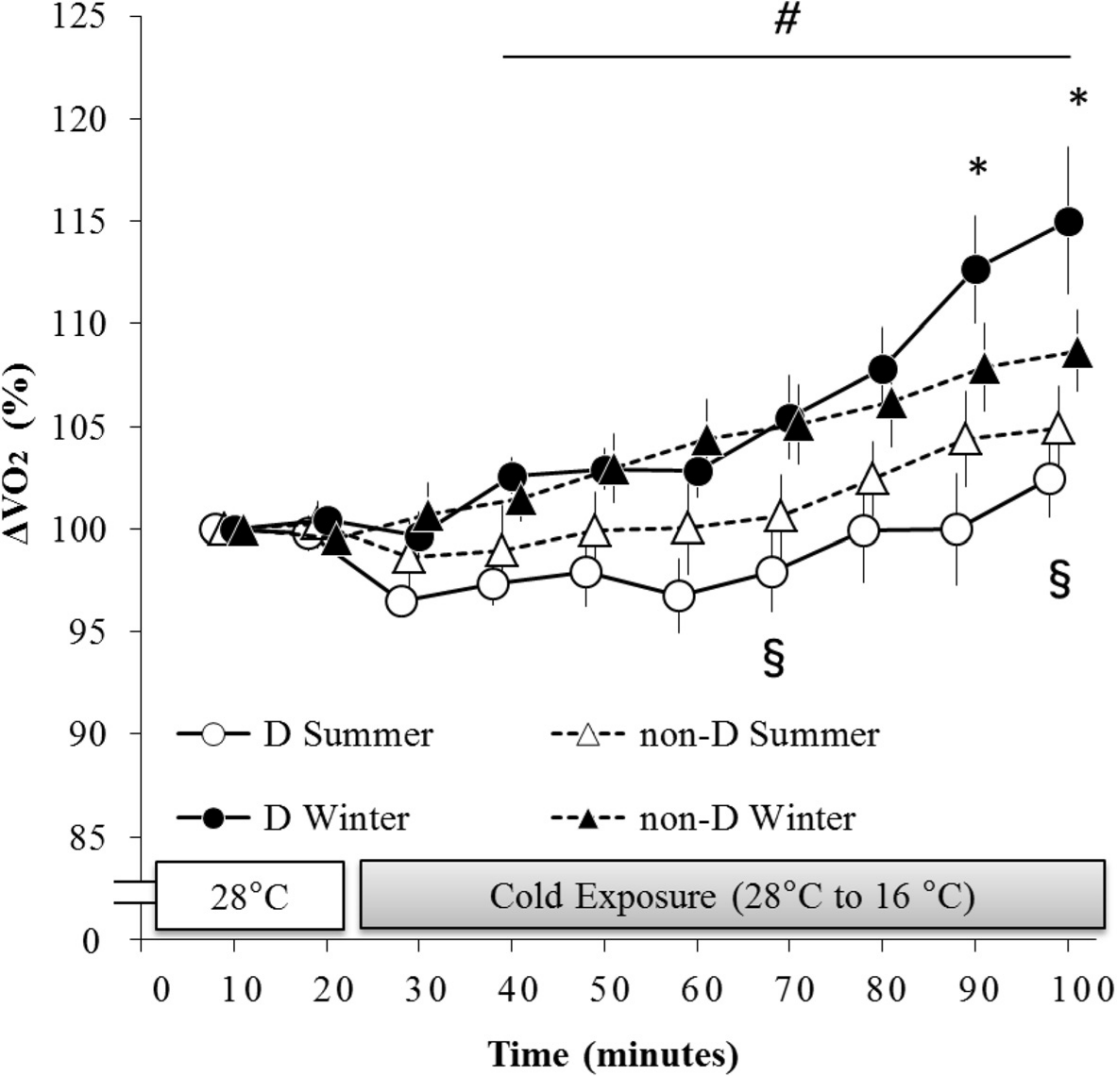
**Changes in VO**_**2 **_**(ΔVO**_**2**_**).** White circles connected by the solid line indicate summer data associated with haplogroup D (n = 8) and white triangles connected by the dotted line indicate summer data associated with haplogroup non-D (n = 9). Black circles connected by the solid line indicate winter data associated with haplogroup D (n = 8) and black triangles connected by the dotted line indicate winter data associated with haplogroup non-D (n = 9). ΔVO_2_ of haplogroup D was significantly higher than that of haplogroup non-D during the period ranging from 90 to 100 minutes in winter. ΔVO_2_ of haplogroup D was significantly higher during the period ranging from 40 to100 minutes in winter than it was during that same period in summer. ΔVO_2_ of haplogroup non-D was significantly higher at both 70 minutes and 100 minutes in winter than it was at those same intervals in summer. *P < 0.05, comparisons between haplogroups D and non-D in winter. *#P* < 0.05, comparisons between haplogroup D values taken in summer and winter. §*P* < 0.05, comparisons between haplogroup non-D values taken in summer and winter.

The main effects of season (F (1, 15) = 17.07, *P* < 0.001) and time (F (9, 135) = 12.10, *P* < 0.001) were significant for ΔVO_2_ (Figure 1). There were also significant interactions between season and group (F (1, 15) = 5.16, *P* < 0.001), season and time (F (9, 135) = 8.96, *P* < 0.001), and group, season, and time (F (9, 135) = 3.23, *P* < 0.005).

In a *post-hoc* test carried out in winter, **Δ**VO_2_ of haplogroup D was significantly greater during the period ranging from 90 to 100 minutes compared with **Δ**VO_2_ of haplogroup non-D during that same period. **Δ**VO_2_ of haplogroup D was significantly greater during the period ranging from 40 to 100 minutes in winter than it was during that period in summer. **Δ**VO_2_ of haplogroup non-D was significantly greater at 70 minutes and 100 minutes in winter than it was at those same points in summer.

### Change in electromyogram (EMG)

**Figure 2:**
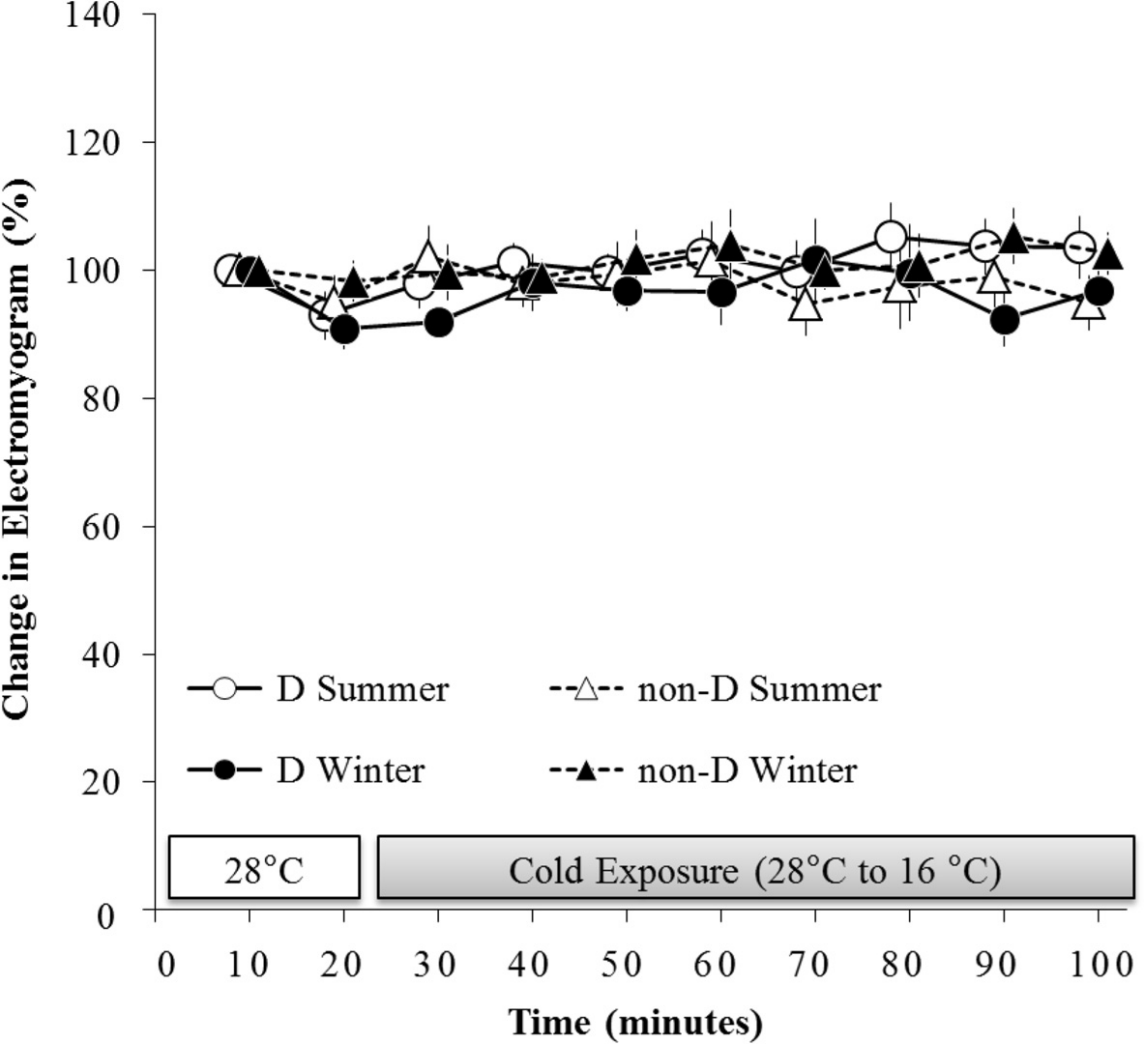
**Changes in electromyogram (EMG).** No significant differences existed between season and group.

Electromyographic data exhibited no significant main effects for season or time, and no significant interaction was observed between season and time (Figure 2).

### Respiratory exchange ratio (RER)

**Figure 3:**
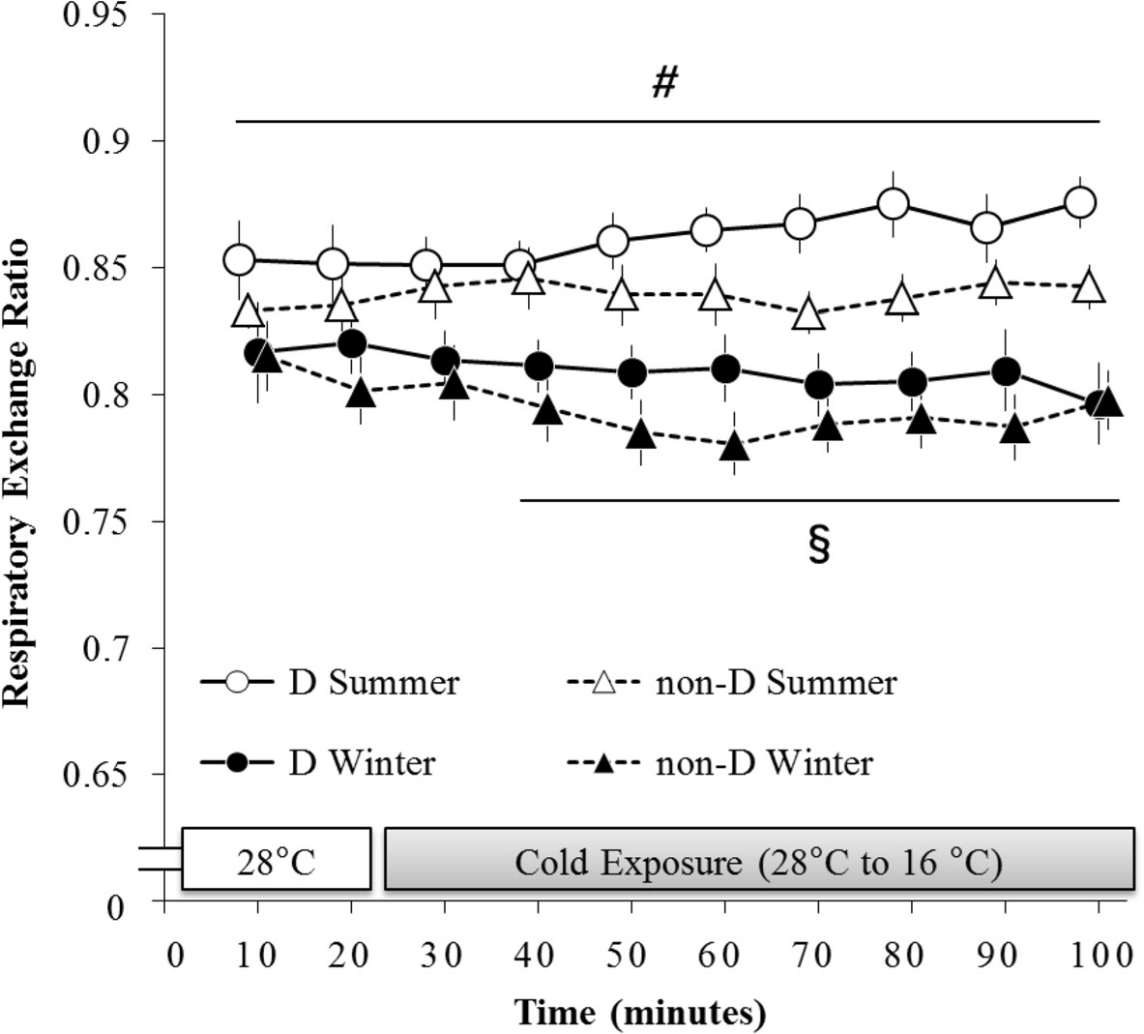
**Changes in respiratory exchange ratio (RER).** RER of haplogroup D was significantly lower during the period ranging from 0 to –100 minutes in winter than it was during that same period in summer. RER of haplogroup non-D was significantly lower during the period ranging from 40 to 100 minutes in winter than it was during that same period in summer. *#P* < 0.05, comparisons between haplogroup D values taken in summer and winter. §*P* < 0.05, comparisons between haplogroup non-D values taken in summer and winter.

The main effect of season (F (1, 15) = 18.22, *P* < 0.001) was significant for RER (Figure 3). Significant interactions were also detected between season and time (F (9, 135) = 5.07, *P* < 0.001), and group, season, and time (F (9, 135) = 2.04, *P* < 0.05).

In a *post-hoc* test, RER of haplogroup D was significantly lower during the period ranging from 0 to 100 minutes in winter than it was during that same period in summer. RER of haplogroup non-D was significantly lower during the period ranging from 30 to 100 minutes in winter than it was during that same period in summer. Despite these seasonal differences, no significant differences in RER were detected between groups.

### Rectal temperature (T_re_)

**Figure 4:**
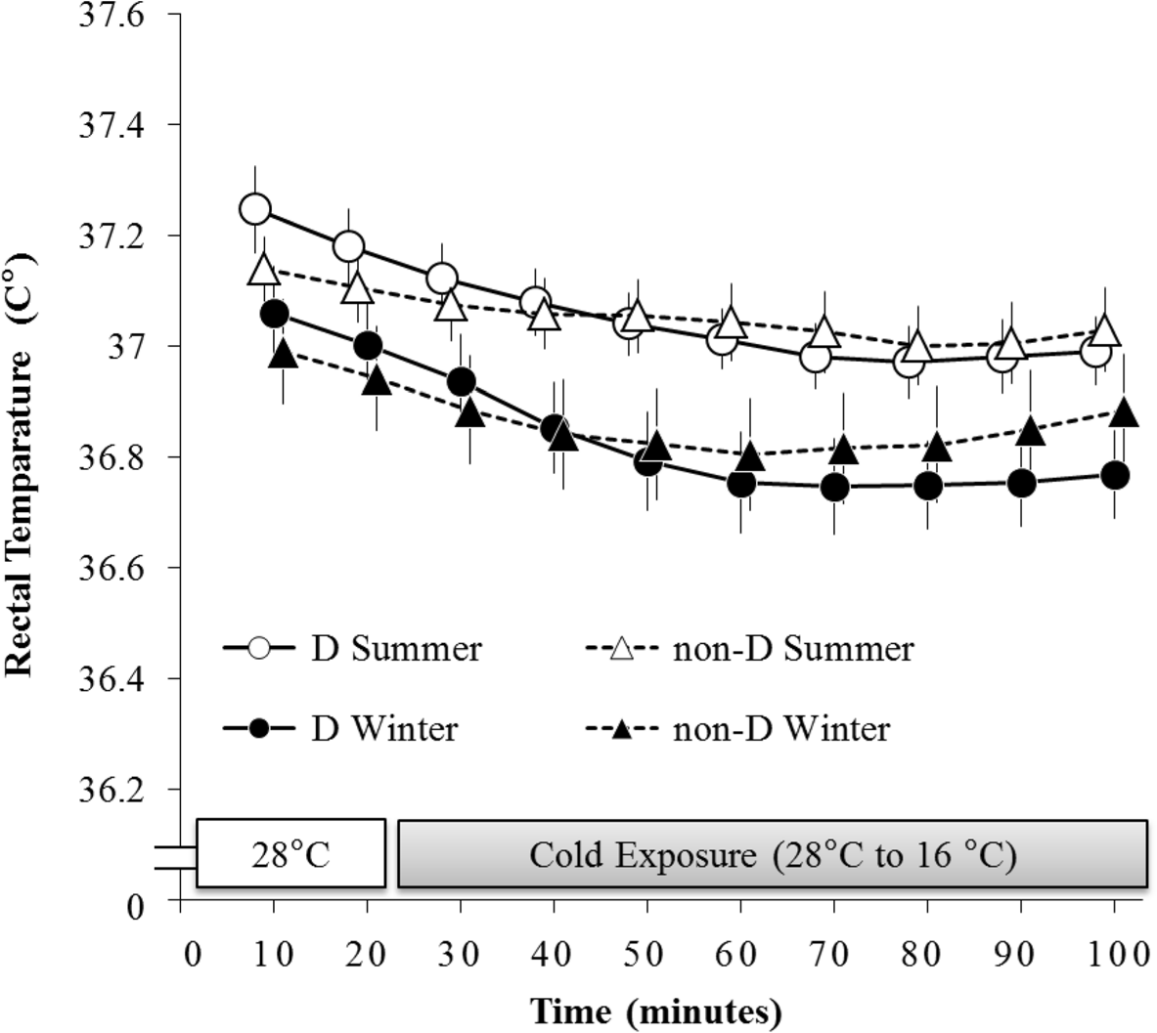
**Changes in rectal temperature.** T_re_ of both the D and non-D groups were significantly lower during the period ranging from 0 to 100 minutes in winter than they were during that same period in summer.

The main effect of time (F (9, 135) = 39.73, *P* < 0.001) was significant for T_re_ (Figure 4). A significant interaction was also detected between season and time (F (9, 135) = 2.33, *P* < 0.05). In a *post-hoc* test conducted using both groups, T_re_ was significantly lower during the period ranging from 0 to 100 minutes in winter than it was during that same period in summer.

### Distal skin temperature ( ${\bar{T}}_{\mathrm{dist}}$)

**Figure 5:**
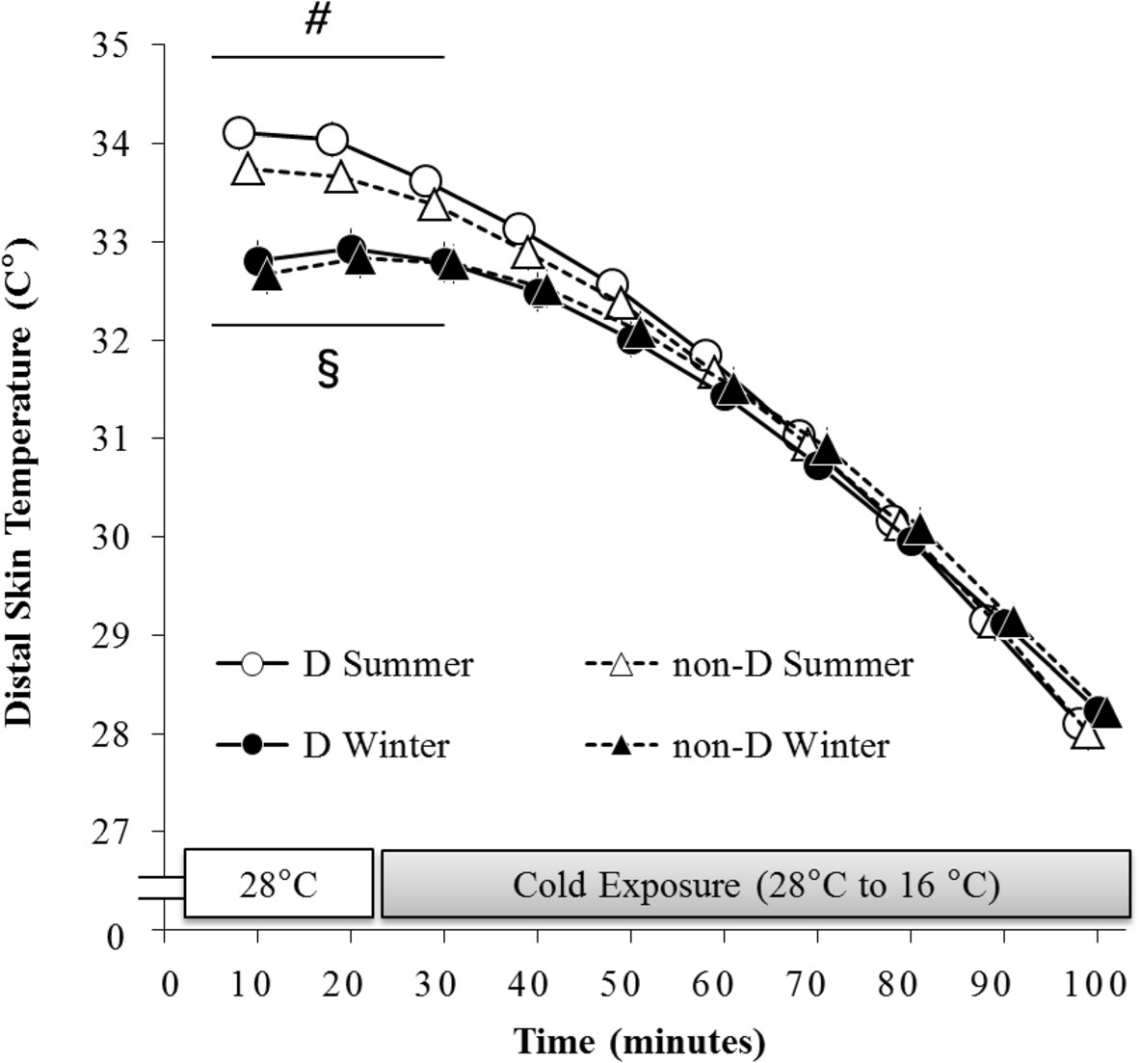
**Changes in distal skin temperature (**${\bar{T}}_{\mathrm{dist}}$**).**${\bar{T}}_{\mathrm{dist}}$ of both the D and non-D groups were significantly lower during the period ranging from 0 to –30 minutes in winter than they were during that same period in summer. *#P* < 0.05, comparisons between haplogroup D values taken in summer and winter. §*P* < 0.05, comparisons between haplogroup non-D values taken in summer and winter.

The main effect of time (F (9, 135) = 2,049.64, *P* < 0.001) was significant for ${\bar{T}}_{\mathrm{dist}}$ (Figure 5). A significant interaction was also detected between season and time (F (9, 135) = 29.84, *P* < 0.001). In a *post-hoc* test conducted using both groups, ${\bar{T}}_{\mathrm{dist}}$ was significantly lower during the period ranging from 0 to 30 minutes in winter than it was during that same period in summer.

## Discussion

In the present study, **Δ**VO_2_ significantly and rapidly increased without shivering in response to cold exposure during winter (Figures 1 and 2). The **Δ**VO_2_ of haplogroup D in particular was significantly greater than that of haplogroup non-D in winter. Furthermore, in a result similar to that of our previous study [21], RER of both haplogroups were significantly lower during periods of rest and periods of cold exposure in winter than they were in summer (Figure 3). These results suggested that NST stimulated by cold was enhanced in winter, and that haplogroup D exhibited greater NST than did haplogroup non-D in winter.

Recent studies suggested that the heat produced by NST is primarily generated by BAT [12,24]. The greater NST of haplogroup D observed in winter might have indicated that the group exhibited greater BAT activity than did haplogroup non-D. Tanaka *et al*. [20] reported that haplogroup D exhibited resistance to metabolic syndrome, which indicated that the greater NST of haplogroup D might have been related to that group’s ability to metabolize fat; however, in the present study, no significant difference in fat metabolism as estimated by RER was detected between groups in winter. These results indicated that total heat generated by NST could not be attributed to BAT alone, and that other factors might have contributed to the greater NST observed in haplogroup D.

Previous studies have reported that mtDNA polymorphism affects replication of mitochondria [14]. BAT, as well as skeletal muscle and liver, are mitochondria-rich tissues. Replication of mitochondria is activated by stimuli such as cold, exercise, and thyroid hormone [25]; therefore, the greater NST observed in haplogroup D was likely the result of seasonal cold acclimatization and an increase in mitochondria. BMR (Basal Metabolic Rate) of Japanese individuals has been shown to exhibit seasonal variation, with higher values in winter than in summer [26]. Fat metabolism in Japanese individuals has also been shown to exhibit seasonal variation, being greater in winter than it is in summer [26]. Results such as these have led previous studies to attribute seasonal metabolic changes to changes in mitochondrial function or activity of NST via BAT or other tissues. Although the source of heat generation in NST remains unclear, it can be concluded that mtDNA haplogroup is a genetic factor that significantly affects **Δ**VO_2_.

Despite its impact on **Δ**VO_2_, mtDNA haplogroup did not affect T_re_ (Figure 4) or ${\bar{T}}_{\mathrm{dist}}$ (Figure 5). Our previous study reported greater values of T_re_ associated with haplogroup D during periods of severe cold exposure (10°C) in summer; however, decreases in T_re_ observed in the present study were comparatively lower than those observed in our previous study due to the fact that only mild cold exposure was employed. Therefore, the present study concluded that there existed no significant effect of mtDNA haplogroup on core body temperature. Furthermore, there was no significant effect of haplogroup on ${\bar{T}}_{\mathrm{dist}}$ in either the present study or our previous study. These results suggested that mitochondrial polymorphism primarily affected thermogenesis involving NST. A similar study employing a more severe or longer cold exposure may allow the effect of mitochondrial polymorphism on body temperature to be elucidated.

**Figure 6:**
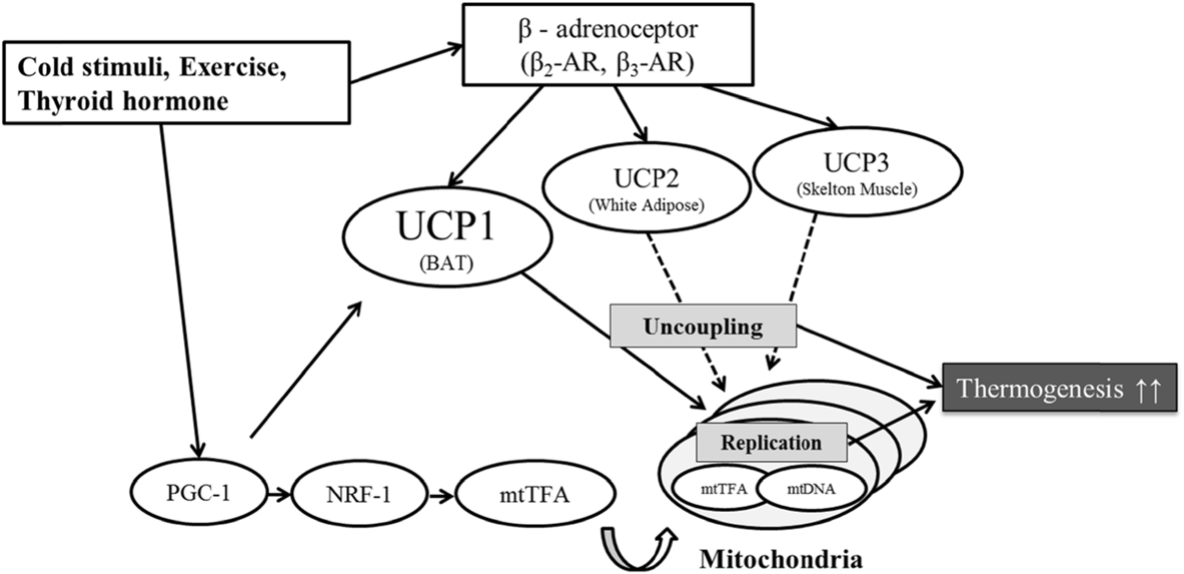
**Physiological cascade associated with human non-shivering thermogenesis (NST).** NST in humans is activated by a complex physiological cascade. Cold stimuli activate gene expression of *PGC-1alpha* (*Peroxisome proliferator-activated receptor-gamma coactivator-1alpha*), *NRF-1* (*Nuclear respiratory factor 1*)*,* and *mtTFA* (*Mitochondrial transcription factor A*), which in turn promote mitochondrial replication. Due to this process, mitochondrial density increases and NST is enhanced. Cold stimuli also enhance *β*_*2*_*-AR* (*beta-2 adrenergic receptor*) and *β*_*3*_*-AR* (*beta-3 adrenergic receptor*) via the sympathetic nervous system activating the expression of *UCP1, UCP2,* and *UCP3*. This increase in *UCP* also enhances thermogenesis. These flows interact with one other, and each associated gene possesses SNPs that affect physiological function.

One problem existed in that mtDNA haplogroup reflected population structure, and significant differences in VO_2_ might have been dependent on other sources of genetic variation. More specifically, differences in NST were affected not only by mitochondrial polymorphism, but also by polymorphisms in other genes, such as *UCP1* and *UCP3.* This dependency was due to the fact that NST is activated by a complex physiological cascade (Figure 6). Hancock *et al*. [27] reported that the genetic diversity of *UCP1* and *UCP3* was shaped by cold climatic conditions, as both genes were related to human energy expenditure involving NST. Polymorphism of *UCP1* affects expression of *UCP1* in BAT [28], and polymorphism of *UCP3* is related to expression of certain proteins in skeletal muscle [29]. Since previous studies suggested that NST was more directly affected by the function of *UCP*, mtDNA haplogroup D and other mitochondrial groups might have co-evolved with other gene polymorphisms. These results illustrated the need to develop a more thorough understanding regarding differences in NST, and future studies should focus on the investigation of more gene polymorphisms related to NST.

The present study was limited by its small sample size, which prevented the influence of genetic factors from being excluded, its lack of direct measurement of BAT, and the fact that it did not measure BMR, which is known to affect human cold adaptability. It is also necessary for future studies to investigate more gene polymorphisms related to human cold adaptation, such as *UCP1-3*. Future studies will require greater numbers of participants, measurement of additional physiological parameters, and will need to investigate more gene polymorphisms.

### Perspective

In order to clarify the relationship between physiological polytypism and gene polymorphism with respect to cold adaptation, not only is it necessary to make additional measurements using methodology similar to that of the present study, it is also necessary to develop a new investigational approach. Most importantly, multiple candidate genes related to human cold adaptation should be examined. For example, in the physiological cascade associated with human NST, there exist important genes that either enhance or depress thermogenesis. Candidate genes with the potential to impact cold adaptation or thermogenesis should be narrowed down through population genetics using methodology similar to that of Hancock *et al*. [27]. Investigation into the effects of candidate genes linked to obesity, BMI, or body fat is also necessary, as these phenotypes are believed to be related to energy expenditure. Nakayama *et al*. [30] previously identified *TRIB2 (tribbles pseudokinase 2)*, which is related to visceral fat and obesity. Their results may have also suggested that gene polymorphism of *TRIB2* was shaped by cold climatic conditions in East Asian populations. Nakayama *et al*. [31] also reported that polymorphism of *UCP1* was related to seasonal variation in visceral fat. Research methods such as these are necessary in order to develop an understanding of the relationship between physiological polytypism and gene polymorphism. In order to further advance our understanding of heat generation involving NST, population genetics and field research must identify genes that are potentially linked to human cold adaptation.

Researchers in the field of physiological anthropology should collaborate with researchers in the disciplines mentioned above in order to accumulate physiological data with respect to adaptation. In addition, sample sizes of physiological experiments should be increased to allow for more a robust discussion, and sampling from various regions should be employed to account for variations in the physical environment such as temperature and day length. In conclusion, due to the importance of genetic research in physiological anthropology, collaboration between population geneticists, field researchers, and physiological researchers will be required in future studies.

## Abbreviations

ANOVA: analysis of variance; BAT: brown adipose tissue; BMI: body mass index; BMR: basal metabolic rate; BSA: body surface area; EMG: electromyogram; FFA: free fatty acids; LCT: lower critical temperature; NST: non-shivering thermogenesis; OXFOS: oxidative phosphorylation; PCR: polymerase chain reaction; RER: respiratory exchange ratio; SNP: single nucleotide polymorphism; ST: shivering thermogenesis; ${\bar{T}}_{\mathrm{dist}}$: distal skin temperature; T_re_: rectal temperature; VCO_2_: carbon dioxide output; VO_2_: oxygen intake; ΔVO_2_: changes in VO_2_; VO_2max_: maximum oxygen intake.

## Competing interests

The authors declare that they have no competing interests.

## Authors’ contributions

TN carried out the design of the present study and data analysis, and drafted the manuscript. SW contributed to the design of the experiments and checked the manuscript. Both authors read and approved the final manuscript.

## References

[B1] Relethford JH Genetic evidence and the modern human origins debate Heredity 2008 100 555 563 10.1038/hdy.2008.14 18322457

[B2] Steegmann AT Pearl Memorial Lecture. Human cold adaptation: an unfinished agenda Am J Hum Biol 2007 227 218 227 17286254 10.1002/ajhb.20614

[B3] Hart JS Sabean HB Hildes JA Depocas F Hammel HT Andersen KL Irving L Foy G Thermal and metabolic responses of coastal Eskimos during a cold night J Appl Physiol 1962 17 953 960 13953036 10.1152/jappl.1962.17.6.953

[B4] Scholander PF Hammel HT Hart JS Lemessurier DH Steen J Cold adaptation in Australian aborigines J Appl Physiol 1958 13 211 218 13575330 10.1152/jappl.1958.13.2.211

[B5] Maeda T Perspectives on environmental adaptability and physiological polymorphism in thermoregulation J Physiol Anthropol Appl Human Sci 2005 24 237 240 10.2114/jpa.24.237 15930813

[B6] Davis TR Johnston DR Seasonal acclimatization to cold in man J Appl Physiol 1961 16 231 234 13720245 10.1152/jappl.1961.16.2.231

[B7] Inoue Y Nakao M Ueda H Araki T Seasonal variation in physiological responses to mild cold air in young and older men Int J Biometeorol 1995 38 131 136 10.1007/BF01208489 7744527

[B8] Mäkinen TM Pääkkönen T Palinkas LA Rintamäki H Leppäluoto J Hassi J Seasonal changes in thermal responses of urban residents to cold exposure Comp Biochem Physiol A Mol Integr Physiol 2004 139 229 238 10.1016/j.cbpb.2004.09.006 15528172

[B9] Maeda T Sugawara A Fukushima T Higuchi S Ishibashi K Effects of lifestyle, body composition, and physical fitness on cold tolerance in humans J Physiol Anthropol Appl Human Sci 2005 24 439 443 10.2114/jpa.24.439 16079594

[B10] Yasukouchi A Yamasaki K Iwanaga K Fujiwara M Sato H Seasonal effects on the relationships between morphological characteristics and decrement of rectal temperature in a cold environment Ann Physiol Anthropol 1983 2 39 44 In Japanese with English abstract 10.2114/ahs1983.2.39

[B11] van Marken Lichtenbelt WD Vanhommerig JW Smulders NM Drossaerts JM Kemerink GJ Bouvy ND Schrauwen P Teule GJ Cold-activated brown adipose tissue in healthy men N Engl J Med 2009 360 1500 1508 10.1056/NEJMoa0808718 19357405

[B12] Saito M Okamatsu-Ogura Y Matsushita M Watanabe K Yoneshiro T Nio-Kobayashi J Iwanaga T Miyagawa M Kameya T Nakada K Kawai Y Tsujisaki M High incidence of metabolically active brown adipose tissue in healthy adult humans: effects of cold exposure and adiposity Diabetes 2009 58 1526 1531 10.2337/db09-0530 19401428 PMC2699872

[B13] van der Lans AA Hoeks J Brans B Vijgen GH Visser MG Vosselman MJ Hansen J Jörgensen JA Wu J Mottaghy FM Schrauwen P, van Marken Lichtenbelt WD: **Cold acclimation recruits human brown fat and increases nonshivering thermogenesis** J Clin Invest 2013 123 3395 3403 10.1172/JCI68993 23867626 PMC3726172

[B14] Wallace DC A mitochondrial paradigm of metabolic and degenerative diseases, aging, and cancer: a dawn for evolutionary medicine Annu Rev Genet 2005 39 359 407 10.1146/annurev.genet.39.110304.095751 16285865 PMC2821041

[B15] van Marken Lichtenbelt WD Schrauwen P Implications of nonshivering thermogenesis for energy balance regulation in humans Am J Physiol Regul Integr Comp Physiol 2011 301 285 296 10.1152/ajpregu.00652.2010 21490370

[B16] Mishmar D Ruiz-Pesini E Golik P Macaulay V Clark AG Hosseini S Brandon M Easley K Chen E Brown MD Sukernik RI Olckers A Wallace DC Natural selection shaped regional mtDNA variation in humans Proc Natl Acad Sci U S A 2003 100 171 176 10.1073/pnas.0136972100 12509511 PMC140917

[B17] Balloux F Handley LJ Jombart T Liu H Manica A Climate shaped the worldwide distribution of human mitochondrial DNA sequence variation Proc Biol Sci 2009 276 3447 3455 10.1098/rspb.2009.0752 19586946 PMC2817182

[B18] Marcuello A Martínez-Redondo D Dahmani Y Casajús JA Ruiz-Pesini E Montoya J López-Pérez MJ Díez-Sánchez C Human mitochondrial variants influence on oxygen consumption Mitochondrion 2009 9 27 30 10.1016/j.mito.2008.10.002 18952007

[B19] Mikami E Fuku N Takahashi H Ohiwa N Scott RA Pitsiladis YP Higuchi M Kawahara T Tanaka M Mitochondrial haplogroups associated with elite Japanese athlete status Br J Sports Med 2011 45 1179 1183 10.1136/bjsm.2010.072371 20551160

[B20] Tanaka M Takeyasu T Fuku N Li-Jun G Kurata M Mitochondrial genome single nucleotide polymorphisms and their phenotypes in the Japanese Ann N Y Acad Sci 2004 1011 7 20 10.1196/annals.1293.002 15126279

[B21] Nishimura T Motoi M Niri Y Hoshi Y Kondo R Watanuki S Relationship between seasonal cold acclimatization and mtDNA haplogroup in Japanese J Physiol Anthropol 2012 31 22 10.1186/1880-6805-31-22 22929588 PMC3443646

[B22] Kurazumi Y Tsuchikawa T Kakutani K Torii T Matsubara N Horikoshi T Evaluation of the conformability of the calculation formula for the body surface area of the human body Jpn J Biometeorol 2009 39 101 106

[B23] Hardy JD DuBois EF The technique of measuring radiation and convection J Nutr 1938 5 461 475

[B24] Yoneshiro T Aita S Matsushita M Kameya T Nakada K Kawai Y Saito M Brown adipose tissue, whole-body energy expenditure, and thermogenesis in healthy adult men Obesity 2011 19 13 16 10.1038/oby.2010.105 20448535

[B25] Irrcher I Adhihetty PJ Sheehan T Joseph AM Hood DA PPARgamma coactivator-1alpha expression during thyroid hormone- and contractile activity-induced mitochondrial adaptations Am J Physiol Cell Physiol 2003 284 1669 1677 10.1152/ajpcell.00409.2002 12734114

[B26] Osiba S The seasonal variation of basal metabolism and activity of thyroid gland in man Jpn J Physiol 1957 7 355 365 13501954 10.2170/jjphysiol.7.355

[B27] Hancock AM Clark VJ Qian Y Di Rienzo A Population genetic analysis of the uncoupling proteins supports a role for UCP3 in human cold resistance Mol Biol Evol 2011 28 601 614 10.1093/molbev/msq228 20802238 PMC3002247

[B28] Su AI Wiltshire T Batalov S Lapp H Ching KA Block D Zhang J Soden R Hayakawa M Kreiman G Cooke MP Walker JR Hogenesch JB A gene atlas of the mouse and human protein-encoding transcriptomes Proc Natl Acad Sci U S A 2004 101 6062 6067 10.1073/pnas.0400782101 15075390 PMC395923

[B29] Schrauwen P Xia J Bogardus C Pratley RE Ravussin E Skeletal muscle uncoupling protein 3 expression is a determinant of energy expenditure in Pima Indians Diabetes 1999 48 146 149 10.2337/diabetes.48.1.146 9892236

[B30] Nakayama K Ogawa A Miyashita H Tabara Y Igase M Kohara K Miki T Kagawa Y Yanagisawa Y Katashima M Onda T Okada K Fukushima S Iwamoto S Positive natural selection of TRIB2, a novel gene that influences visceral fat accumulation, in East Asia Hum Genet 2013 132 201 217 10.1007/s00439-012-1240-9 23108367

[B31] Nakayama K Miyashita H Yanagisawa Y Iwamoto S Seasonal effects of UCP1 gene polymorphism on visceral fat accumulation in Japanese adults PLoS One 2013 25 e74720 24086366 10.1371/journal.pone.0074720 PMC3783463

